# The estimation of a preference-based single index for the IBS-QoL by mapping to the EQ-5D-5L in patients with irritable bowel syndrome

**DOI:** 10.1007/s11136-021-02995-y

**Published:** 2021-09-21

**Authors:** Rosel Sturkenboom, Daniel Keszthelyi, Lloyd Brandts, Zsa Zsa R. M. Weerts, Johanna T. W. Snijkers, Ad A. M. Masclee, Brigitte A. B. Essers

**Affiliations:** 1 Division of Gastroenterology-Hepatology, Department of Internal Medicine, NUTRIM School for Nutrition and Translational Research in Metabolism, Maastricht University Medical Center, P.O. Box 5800, 6202 AZ Maastricht, The Netherlands; 2 Department of Clinical Epidemiology and Medical Technology Assessment, CAPHRI Care and Public Health Research Institute, Maastricht University Medical Center, Maastricht, The Netherlands

**Keywords:** Mapping, EQ-5D-5L, IBS-QoL, Irritable bowel syndrome, Utility, Quality of life

## Abstract

**Purpose:**

The Irritable Bowel Syndrome Quality of Life (IBS-QoL) questionnaire is a commonly used and validated IBS-specific QoL instrument. However, this questionnaire is in contrast to the EQ-5D-5L, not preference-based and as such does not allow calculation of QALYs. The objective of this study was to describe the convergent- and known-group validity of both questionnaires and to develop a mapping algorithm from EQ-5D-5L which enable IBS-QoL scores to be transformed into utility scores for use in economic evaluations.

**Methods:**

We used data from two multicenter randomized clinical trials, which represented the estimation and external validation dataset. The convergent validity was investigated by examining correlations between the EQ-5D-5L and IBS-QoL and the known-group validity by calculating effect sizes. Ordinary least squares (OLS), censored least absolute deviations (CLAD), and mixture models were used in this mapping approach.

**Results:**

283 IBS patients were included (*n* = 189 vs. *n* = 84). Mean IBS-QoL score was 71.13 (SD 15.66) and mean EQ-5D-5L utility score was 0.73 (SD 0.19). The overall sensitivity of the IBS-QoL and EQ-5D-5L to discriminate between patient and disease characteristics was similar. CLAD model 4, containing the total IBS-QoL score and squared IBS-SSS (IBS severity scoring system), was chosen as the most appropriate model to transform IBS-QoL scores into EQ-5D-5L utility scores.

**Conclusion:**

This study reports the development of an algorithm where the condition-specific questionnaire IBS-QoL can be used to calculate utility values for use in economic evaluations. Including a clinical measure, IBS-SSS, in the model improved the performance of the algorithm.

**Supplementary Information:**

The online version contains supplementary material available at 10.1007/s11136-021-02995-y.

## Introduction

Irritable bowel syndrome (IBS) is a chronic disorder of the gut–brain interaction characterized by altered bowel habits (constipation, diarrhea, or mixed pattern) and abdominal pain. IBS affects a large number of people worldwide, 4.4–4.8% according to the Rome IV criteria [1]. These symptoms have a substantial impact on patients’ quality of life (QoL) and are associated with considerable use of healthcare resources and secondary significant economic impact on individuals, healthcare systems, and society. Between 15 and 50% of patients with IBS report absenteeism (work time missed) due to their symptoms and up to 34% report presenteeism (impairment while at work) [2, 3]. To accomplish symptom control to improve quality of life (QoL), various treatments for IBS are available nowadays. These include diets, psychological interventions, and several types of pharmacological agents [4, 5].

The cost-effectiveness of these treatments is generally examined using cost-utility analysis [6, 7]. In health care decision-making and reimbursement procedures, the outcome of a cost-utility analysis is known as quality-adjusted life years (QALY) which is used to determine whether a new therapy delivers value for money [8]. The quality of life side of the QALY can be assessed with a generic questionnaire, such as the frequently used EQ-5D (European Quality of Life Five Dimension questionnaire), which is designed to cover the core dimensions of health that are relevant across all medical conditions and to allow comparisons between patient groups [9, 10]. In order to capture the impact of IBS on QoL, patients complete the EQ-5D-3L or the recently developed 5-level questionnaire after which a value set is applied to generate utility values. For example, mean-utility values in IBS patients range between 0.50 and 0.75, where 1 is equivalent to perfect health and 0 is death [3, 8, 11–14]. The utility scores are subsequently used to calculate QALYs [8]. However, in clinical studies, a non-preference-based condition-specific questionnaire is often preferred because they capture more disease-specific or relevant aspects of the disease from a clinical and patients’ perspective. The Irritable Bowel Syndrome Quality of Life questionnaire (IBS-QoL) is a condition-specific instrument for IBS patients which incorporates specific subdomains such as food avoidance, bowel habits, and the effect on the social/sexual relationships [15]. EuroQoL-5D and IBS-QoL have both been proven to be valid for assessing QoL in IBS patients [3, 8, 15]. However, previous studies, in which different disease populations were examined, have suggested that condition-specific measures are more responsive than the generic measure with regard to capturing changes in health [16–19]. The involvement of the psychological domain in QoL questionnaires is relevant for IBS patients, due to the high prevalence of anxiety and depression disorders among these category of patients which has a significant impact on the disease course and the choice of therapy [2, 12, 20–23]. The EQ-5D-5L has one Anxiety/Depression dimension, where IBS-QoL has several domains containing psychological questions. Whether the general EQ-5D and the condition-specific IBS-QoL are both sensitive enough to capture (mental) health changes is not yet investigated in IBS patients. Therefore, the difference in responsiveness of both the EQ-5D-5L and IBS-QoL should be further explored.

Because the IBS-QoL is specifically designed for IBS patients and uses aspects that are salient to this specific patient group, the IBS-QoL is often preferred in clinical studies. Up to now, however, there is no proper method available to convert IBS-QoL scores into utilities to calculate QALYs. A mapping approach for the IBS-QoL to the EQ-5D-5L would be highly valuable to enable prediction of utility scores for modeling studies in which evidence is used from trials where in the past, only the IBS-QoL questionnaire is included [24]. Mapping is recognized by the National Institute for Health and Clinical Excellence (NICE) for generating utility information for non-preferences-based measures and the ISPOR (International Society for Pharmacoeconomics and Outcomes Research) guidelines have provided recommendations about this composed algorithm between a base measure and a target measure [25–27].

To the best of our knowledge, no study thus far has performed a mapping approach to predict utility values for the condition-specific measure IBS-QoL for use in IBS patients. The goal of this study is to examine the convergent- and known-group validity between the EQ-5D-5L and the IBS-QoL and use empirical mapping to predict EQ-5D-5L utility values from the non-preference-based measure IBS-QoL scores in IBS patients.

## Method

### Datasets

Two studies were included for this mapping approach. The first study (*N* = 189) is a three-armed multicenter placebo-controlled randomized controlled trial where the efficacy of peppermint oil was assessed, the PERSUADE study (NCT02716285) [28]. Patient inclusion took place in the Netherlands from August 2016 through March 2018. This study was used as estimation data set to create the mapping algorithm.

The second study (*N* = 84) is a three-armed multicenter randomized controlled non-inferiority trial where the efficacy of online hypnotherapy versus face-to-face hypnotherapy is compared with online psychoeducation as control condition (FORTITUDE NCT03899779). Patient inclusion commenced in the Netherlands in July 2019 and is still ongoing. This trial was used as study data set to test the algorithm for external validation.

Inclusion criteria were similar in both studies. Subjects were included between age 16 and 75 years, diagnosed with IBS according to the Rome IV criteria, and had no history of other causes for the abdominal complaints, such as Crohn’s disease and coeliac disease [29]. They were both recruited via primary and secondary/tertiary healthcare. There was a slight difference in the age limits for inclusion between both studies: in the estimation data set, subjects between 18 and 75 years of age are included, in the validation set, subjects were included with 16–65 years of age. This is due to the changed age limit by the Dutch Medical Research Involving Human Subjects Act in august 2016 where research is allowed with subjects from 16 years and older [30]. The upper limit of age was adjusted due to involvement of online therapies. Exclusion criteria of both trials included insufficient command of the Dutch language, major surgery to the lower gastrointestinal tract, current pregnancy or lactation, and, respectively, peppermint oil usage or hypnotherapy in the last 3 months prior to inclusion. Patients with a positive screening for anxiety and depression (score ≥ 10 of GAD-7 and PHQ-9, respectively) in the validation dataset were interviewed by the researcher and only patients with clinically significant anxiety or depression were excluded. In the estimation dataset, these scores were not incorporated during patient screening.

All procedures were in accordance with the ethical standards of the responsible committee on human experimentation (institutional and national) and with the Helsinki Declaration of 1964, as revised in 2013. Both studies were reviewed and approved by the ethics committee at the Maastricht University Medical Center (METC 162,009; METC 18–037). Informed consent was obtained from all patients prior to being included in the study.

### Questionnaires

Both the EQ-5D-5L and IBS-QoL were completed in these studies. The EQ-5D-5L is a preference-based measure and consists of five-dimension mobility, self-care, usual activities, pain/discomfort, and anxiety/depression, each with five severity levels (no, slight, moderate, severe, extreme problems) [10, 31]. This questionnaire is validated for use in IBS patients [3, 8]. In the Netherlands, it is recommended by the National Health Care Institute (ZIN) for use in cost-utility analyses and a Dutch Tariff for the EQ-5D-5L is applied to create the utility values [32].

The IBS-QoL is a condition-specific instrument that is used to assess the impact of IBS and effects of treatment. It consists of 34 questions which cover eight domains including dysphoria, interference with activity, body image, health worry, food avoidance, social reaction, sexual, and relationships [15, 33]. Each item has a five-point response scale (not at all, slightly, moderately, quite a bit, extremely). The responses are summed and averaged for a total score and transformed to a scale between 0 and 100: higher scores indicating better IBS-specific QoL. The Generalized Anxiety Disorder-7 (GAD-7) [34] and Patient Health Questionnaire-9 (PHQ-9) [35, 36] were completed to screen for anxiety disorders, respectively, depressive disorders. A score of 10 or higher in both questionnaires was considered as cut-off point for (possible) diagnosis of the specific disorder, generalized anxiety, or depression disorder, and further examination to confirm diagnosis is recommended at that point. The Irritable Bowel Syndrome Severity Scoring System (IBS-SSS) was completed to measure the severity of the symptoms (0–500) [37]. It consists of five items with a maximum score of 100; higher scores indicate more severe symptoms.

### Statistical Analysis

Descriptive analyses were performed for patient characteristics. Whether the IBS-QoL and EQ-5D-5L are sensitive to discriminate between relevant disease or patient characteristics was examined by comparing the mean values using paired *t* tests [38]. We hypothesized that both questionnaires would show similar levels of discriminatory power with regard to patient characteristics (age, gender, and BMI). In addition, we hypothesized that the IBS-QoL would have greater discriminatory power for disease characteristics (IBS severity, depression, and anxiety) compared to EQ-5D-5L.

The known-group validity was analyzed using standardized effect sizes, dividing the difference in means by the standard deviation. We used Cohen’s d to calculate the effect size by the pooled standard deviation of the population, where 0.2 was considered as a small effect, 0.5 a medium effect, and 0.8 a large size [39]. If the sample size was small (< 20), Hedges’ g was used to describe the effect size [40]. Glass’ delta was chosen if the variance in both groups significantly differed [41].

The data from both trials were used to estimate a direct response mapping algorithm between IBS-QoL and EQ-5D-5L. The mapping approach was conducted following the principle described by Brazier et al. [42] and the ISPOR guidelines [27]. One of the criteria of mapping is the essential of overlap between the start and target measure to cover the important aspects of HRQoL. Mapping would be unsuccessful if there is no conceptual overlap [26]. At first, convergent validity was investigated by examining the correlations between the paired observations and their domains using Spearman correlation coefficients. Correlation coefficients of 0.10, 0.10–0.50, and > 0.50 were considered as weak, moderate, and strong associations, respectively [43]. Second, different types of regression models were estimated with increasing complexity. As recommended by Brazier et al., our initial analysis included a simple model where the regression consists of the target measure onto the total score of the starting measure (IBS-QoL) [42]. Afterward, the domain scores of the IBS-QoL, whether or not combined with covariates, were added to the algorithm [42]. We tested whether the models improved when including clinical covariates (age, BMI, sex, IBS-subtype, IBS-SSS) [27]. Only age and the clinical variable IBS-SSS significantly improved the models (*p* ≤ 0.05). These two variables were therefore included in the final models, as shown below.

The included models were specified as the following equations:

EQ-5D-5L is the EQ-5D-5L utility score; IBS-QoL is the IBS-QoL total score; Dysphoria score is the score of the domain Dysphoria of the IBS-QoL; Body Image score is the score of the domain Body Image of the IBS-QoL; the IBS-SSS score is the IBS Severity Score (0–500). Also, the squared term of the IBS-SSS is included in the models to capture non-linear effect. *β*_0_ is a constant, *β*_1_, *β*_2_, *β*_3_, are the coefficients to be estimated.

1. Total IBS-QoL score model
  $$ {\text{EQ}} - {\text{5D}} - {\text{5L }} = \beta_{0} + \beta_{{1}} *{\text{ IBS}} - {\text{QoL score}} $$
2. Total IBS-QoL score + IBS-SSS score model + age
  $$ {\text{EQ}} - {\text{5D}} - {\text{5L }} = \beta_{0} + \beta_{{1}} *{\text{ IBS}} - {\text{QoL score }} + \beta_{{2}} *{\text{ IBS}} - {\text{SSS score}} $$
3. Domain Dysphoria score + domain Body Image Score model
  $$ {\text{EQ}} - {\text{5D}} - {\text{5L }} = \beta_{0} + \beta_{{1}} *{\text{ Dysphoria score }} + \beta_{{2}} *{\text{ Body Image score}} $$
4. Total IBS-QoL score + Squared IBS-SSS score model
  $$ {\text{EQ}} - {\text{5D}} - {\text{5L }} = \beta_{0} + \beta_{{1}} *{\text{ IBS}} - {\text{QoL score }} + \beta_{{2}} *{\text{ IBS}} - {\text{SSS score}}^{{2}} $$
5. Dysphoria score + Body image score + Squared IBS-SSS score model + age
  $$ {\text{EQ}} - {\text{5D}} - {\text{5L }} = \beta_{0} + \beta_{{1}} *{\text{ Dysphoria score }} + \beta_{{2}} *{\text{ Body Image score }} + \beta_{{3}} *{\text{ IBS}} - {\text{SSS score}}^{{2}} $$

Overall, EQ-5D-5L utility score is the dependent variable in the different regression equations, while the IBS-QoL total score, the separate domains, and the IBS-SSS score were used as predictors. Three statistical approaches were used to estimate these five models. The first technique was the ordinary least squares (OLS) estimator because it is the most widely used analysis and generates good estimate results, mostly better than the alternatives [26, 44]. It estimates parameters by minimizing the sum of squared errors of data. However, because the utilities of the EQ-5D-5L in our population were censored (skewed left), we investigated the option for using estimators for censoring issues. The censored least absolute deviations (CLAD) estimator was chosen above the Tobit estimator because CLAD is robust against departures of errors from homoskedasticity and normality [45, 46].

The Adjusted Limited Dependent Variable Mixture Model (ALDVMM) was used as third mapping model, which was developed to deal with the distributional features of the EQ-5D [47]. It accounts for the gap between 1 (full health) and the highest EQ-5D index value below 1 (truncation point). We used the command aldvmm in Stata to fit these models [48]. First, we estimated the mixture models with two to five components to determine that the model with 4 components has the best fit (highest Likelihood and the lowest BIC (Bayesian information criterion)). Models were conducted with and without inclusion of the truncation point. Model fit was better when the truncation point was included.

Models that were developed using data from one trial were used to predict EQ-5D values in the other trial (external validation). Model fit was assessed by comparing the mean absolute error (MAE) and the root-mean-square error (RMSE) in this sample [27]. The lower the MAE/RMSE, the better the predictive accuracy of the model. A scatter plot of observed and predicted values in the estimation sample was provided of the best model. The best fitting model was selected by the value of MAE/RMSE, the predictive performance, and by the convenience of the algorithm (e.g., simplicity) for usage in clinical practice [49]. Greater complexity of the algorithm by including more clinical and demographic characteristics does not always seem to be beneficial [42]. A significance level of p < 0.05 was applied for all analysis. All analyses were performed in Stata version 14.1 (Stata Corp., College Station, Texas, USA) and IBM SPSS Statistics version 27.0 (Armonk, NY: IBM Corp.).

## Results

### Baseline characteristics of the population

In total, 273 IBS patients were included in this mapping approach. The estimation data set consisted of 189 IBS patients. The external validation data set consisted of 84 patients. The baseline patient characteristics are shown in Table 1. The mean age of the population was 35.07 years and 76.20% was female. The mean IBS-SS score was 278.17 (SD 76.17) which implies a moderate severity of IBS symptoms. The mean quality of life (QoL) according to the general questionnaire EQ-5D-5L was 0.73 (SD 0.20) and the mean QoL according to the condition-specific questionnaire IBS-QoL was 71.13 (SD 15.66).

**Table 1:** Baseline characteristics of the population

	Estimation dataset *N* = 189	Validation dataset *N* = 84	Total *N* = 273
Mean age, years (SD)	34.01 (13.29)	37.44 (13.42)	35.07 (13.39)
Female sex, *n* (%)	147 (77.80)	61 (72.60)	208 (76.20)
Median BMI (kg/m^2^) (SD)	25.57 (5.35)	24.75 (4.73)	25.32 (5.17)
IBS subtype			
Diarrhea (IBS-D), *n* (%)	83 (43.90)	27 (32.10)	110 (40.30)
Constipation (IBS-C), *n* (%)	42 (22.20)	23 (27.40)	65 (23.80)
Mixed (IBS-M), *n* (%)	40 (21.20)	19 (22.60)	59 (21.60)
Undefined (IBS-U), *n* (%)	24 (12.70)	15 (17.90)	39 (14.30)
Mean IBS-SSS score (SD)^a^	276.48 (71.95)	281.98 (85.25)	278.17 (76.17)
Severity of IBS (IBS-SSS)^a^			
Mild IBS, *n* (%)	15 (7.90)	8 (9.50)	23 (8.40)
Moderate IBS, *n* (%)	100 (52.90)	34 (40.50)	134 (49.10)
Severe IBS, *n* (%)	74 (39.20)	42 (50.00)	116 (42.50)
Mean total IBS-QoL score (SD)	73.02 (15.15)	66.88 (16.05)	71.13 (15.66)
Mean total EQ-5D-5L score (SD)	0.73 (0.19)	0.72 (0.21)	0.73 (0.20)
Mean depression score (PHQ-9) (SD)^b^	6.77 (4.55)	5.83 (3.93)	6.48 (4.38)
Depression (PHQ-9)^b^			
Minimal symptoms, *n* (%)	75 (39.70)	38 (45.20)	113 (41.40)
Mild depression, *n* (%)	77 (40.70)	30 (35.70)	107 (39.40)
Moderate depression, *n* (%)	20 (10.60)	13 (15.50)	33 (12.10)
Moderately severe depression, *n* (%)	14 (7.40)	3 (3.60)	17 (6.20)
Severe depression, *n* (%)	3 (1.60)	0	3 (1.10)
Mean anxiety score (GAD-7) (SD)^c^	5.39 (4.35)	4.92 (3.74)	5.25 (4.17)
Anxiety (GAD-7)^c^			
Minimal symptoms, *n* (%)	91 (48.10)	41 (48.80)	132 (48.40)
Mild anxiety, *n* (%)	69 (36.50)	32 (38.10)	101 (37.00)
Moderate anxiety, *n* (%)	17 (9.00)	10 (11.90)	27 (9.90)
Severe anxiety, *n* (%)	12 (6.30)	1 (1.20)	13 (4.80)

^a^The IBS-SSS, IBS symptom severity score, consists of 5 items with a maximum score of 100; a higher score indicates severe IBS symptoms. The total score (range 0–500) can be categorized as Mild IBS (score < 175), Moderate IBS (175–300), and Severe IBS (300–500).

^b^The PHQ-9, Patient Health Questionnaire-9, is a 9-item questionnaire to screen for a depressive disorder. The total score (range 0–27) can be categorized as Minimal symptoms (score 0–4), Mild depression (5–9), Moderate depression (10–14), Moderately severe depression (15–19), Severe depression (20–27).

^c^The GAD-7, Generalized Anxiety Disorder-7, is a 7-item questionnaire to screen for an anxiety disorder. The total score (range 0–21) can be categorized as Minimal symptoms (score 0–4), Mild anxiety (5–9), Moderate anxiety (10–14), Severe anxiety (15–21).

### Convergent- and known-group validity

The convergent validity between the IBS-QoL instrument and the EQ-5D-5L were investigated by Spearman’s correlation coefficient and results are available in Table 2. The correlation between these two instruments for the total score showed a moderately strong significant correlation (0.472). The majority (57.50%) of the correlations between the subscores of the IBS-QoL and the subscores of the EQ-5D-5L were statistically significant. All subscores of the IBS-QoL were positively significantly correlated with the total EQ-5D-5L scores. The subdomains dysphoria (0.420*) and body image (0.438*) of the IBS-QoL reached the strongest significant correlation with the total EQ-5D-5L score.

**Table 2:** Spearman’s correlation coefficients between IBS-QoL values and EQ-5D-5L values

	EQ-5D mobility	EQ-5D selfcare	EQ-5D usual activities	EQ-5D pain	EQ-5D anxiety and depression	Total EQ-5D score
IBS-QoL Dysphoria	− 0.061	0.080	− 0.389*	− 0.204*	− 0.429*	0.420*
IBS-QoL interference with activity	− 0.142	− 0.058	− 0.459*	− 0.159*	− 0.323*	0.378*
IBS-QoL body image score	− 0.106	− 0.012	− 0.354*	− 0.322*	− 0.377*	0.438*
IBS-QoL health worry score	− 0.041	0.080	− 0.277*	− 0.231*	− 0.221*	0.307*
IBS-QoL food avoidance score	− 0.021	− 0.012	− 0.371*	− 0.165*	− 0.258*	0.275*
IBS-QoL social reaction score	0.055	0.154*	− 0.312*	− 0.140	− 0.277*	0.280*
IBS-QoL Sexual score	− 0.097	− 0.033	− 0.226*	− 0.130	− 0.185*	0.224*
IBS-QoL Relationships	− 0.059	0.031	− 0.290*	− 0.089	− 0.328*	0.317*
IBS-QoL overall score	− 0.072	0.040	− 0.482*	− 0.239*	− 0.425*	0.472*

^*^Significance level *p* < 0.05

The analysis of the known-group validity of both HRQoL instruments is shown in Table 3. Both the IBS-QoL and the EQ-5D-5L revealed a similar (very) small non-significant difference in QoL score or utility value with respect to gender (in the estimation set, males have lower health state scores/values, whereas in the validation set, females have lower health state scores/values). Patients younger than 40 years old showed lower quality of life scores or utility values and this effect was significant in the validation set. Greater effect sizes were seen in the validation set compared to the estimation set. This is probably due to a higher mean level of age and the presence of a greater percentage of the subgroup of patients aged ≥ 40 years in the validation dataset. The IBS-QoL score and EQ-5D-5L value were both lower in patients with severe IBS symptoms compared to patients with mild/moderate symptoms (all were significant). The difference in effect sizes between both datasets could be explained by the greater percentage of patients included with mild/moderate symptoms in the estimation dataset compared to the validation dataset. This observation is therefore reflected in the different effect sizes of the depression and anxiety subgroups, whereby patients with more severe symptoms have often more psychopathology. Patients with a depression had significantly lower health scores and values, both according to IBS-QoL and EQ-5D-5L in the validation set. Patients with anxiety also had lower health-related quality of life, according to both instruments.

**Table 3:** Differences in baseline health state values with corresponding effect sizes according to patient and disease characteristics

	Estimation dataset (*N* = 189)								Validation dataset (*N* = 84)					
	IBS-QoL				EQ-5D-5L				IBS-QoL			EQ-5D-5L		
	No. (*n* =)	Mean	*ρ* value	Effect size	Mean	*ρ* value	Effect size	No. (*n* =)	Mean	*ρ* value	Effect size	Mean	*ρ* value	Effect size
Male	42	71.219	*0.458*	− 0.165	0.713	*0.423*	− 0.140	23	69.885	*0.294*	0.252	0.723	*0.951*	0.015
Female	147	73.529			0.741			61	65.743			0.720		
Aged < 40	130	72.115	*0.226*	− 0.191	0.736	*0.847*	0.030	52	63.108	0.003*	− 0.783	0.685	0.032*	− 0.570
Aged ≥ 40	59	75.000			0.730			32	73.001			0.779		
BMI < 25	101	71.717	*0.229*	− 0.177	0.744	*0.623*	0.072	51	67.200	*0.868*	0.038	0.737	*0.390*	0.195
BMI ≥ 25	88	74.392			0.730			32	66.590			0.696		
Mild/moderate symptoms (IBS-SSS < 300)	115	76.113	0.001*	0.473	0.779	0.001*	0.464	42	74.265	< 0.001*	1.032	0.805	< 0.001*	0.861
Severe symptoms (IBS-SSS > 300)	74	68.204			0.666			42	59.489			0.637		
Depression (PHQ-9 ≥ 10)	37	75.219	*0.325*	− 0.181	0.749	*0.624*	− 0.090	16	55.882	0.002*	0.884	0.559	< 0.001*	1.001
No depression (PHQ-9 < 10)	152	72.479			0.731			68	69.464			0.759		
Anxiety (GAD-7 ≥ 10)	29	72.978	*0.936*	− 0.016	0.699	*0.306*	0.207	11	59.492	*0.102*	0.530	0.548	*0.074*	0.609
No anxiety (GAD-7 < 10)	160	73.225			0.741			73	67.989			0.747		

^*^Significance level *p* < 0.05

The IBS-QoL reported greater effect sizes compared to EQ-5D-5L with respect to the characteristics gender (male vs female), age (< 40 vs > 40 years old), and the severity of symptoms according to the IBS-SSS (mild/moderate symptoms vs severe symptoms) and is therefore more sensitive to discriminate here. The discriminatory power of the IBS-QoL and EQ-5D-5L is similar for the BMI score and the presence of depression, but for anxiety, the EQ-5D-5L is slightly more sensitive.

### Mapping results

Data of IBS-QoL and EQ-5D-5L in the estimation dataset were both left-skewed, where the EQ-5D-5L values were bimodally distributed. The EQ-5D-5L values were distributed as follows: 25% of the observations were between -0.02 and 0.68, 25% were between 0.68 and 0.82, 25% were between 0.82 and 0.86, and 25% were between 0.86 and 1.00 (full health). The truncation point for EQ-5D-5L is 0.92.

The goodness of fit results of the five models are shown in Table 4. The MAE ranged from 0.117 to 0.118 and the RMSE from 0.166 to 0.171 for the OLS mapping functions. OLS model 4 performed best, containing the lowest MAE and RMSE. The MAE ranged from 0.111 to 0.114 and the RMSE from 0.168 to 0.191 for the CLAD mapping functions. CLAD model 4 performed best, containing the lowest MAE and RMSE. The MAE ranged from 0.118 to 0.123 and the RMSE from 0.169 and 0.175 for ALDVMM mapping functions. ALDVMM model 4 performed best with the lowest MAE and RMSE.

**Table 4:** Goodness-of-fit results for mapping from IBS-QoL to EQ-5D-5L score

	Observed EQ-5D utility	Predicted EQ-5D utilities				
		Total IBS-QoL score	Total IBS-QoL score + IBS-SSS + age	Dysphoria score + Body image score	Total IBS-QoL score + squared IBS-SSS	Dysphoria score + Body image score + squared IBS-SSS + age
		OLS Model 1	OLS Model 2	OLS Model 3	OLS Model 4	OLS Model 5
Mean (SE)	0.734 (0.014)	0.734 (0.008)	0.734 (0.008)	0.734 (0.008)	0.734 (0.008)	0.734 (0.008)
Minimum	− 0.02	0.387	0.377	0.392	0.360	0.381
Maximum	1.00	0.916	0.936	0.884	0.925	0.914
MAE		0.118	0.118	0.118	0.117	0.117
RMSE		0.170	0.168	0.171	0.166	0.168
Adjusted *R*^2^		0.271	0.296	0.270	0.306	0.295

		CLAD Model 1	CLAD Model 2	CLAD Model 3	CLAD Model 4	CLAD Model 5
Mean (SE)	0.734 (0.014)	0.791 (0.004)	0.777 (0.006)	0.784 (0.006)	0.763 (0.007)	0.797 (0.004)
Minimum	− 0.02	0.591	0.517	0.507	0.415	0.586
Maximum	1.00	0.896	0.925	0.898	0.940	0.887
MAE		0.114	0.111	0.112	0.113	0.114
RMSE		0.185	0.175	0.179	0.168	0.191
Pseudo *R*^2^		0.148	0.155	0.154	0.153	0.163

		ALDVMM Model 1	ALDVMM Model 2	ALDVMM Model 3	ALDVMM Model 4	ALDVMM Model 5
Mean (SE)	0.734 (0.014)	0.744 (0.005)	0.741 (0.004)	0.742 (0.005)	0.745 (0.006)	0.738 (0.006)
Minimum	− 0.02	0.515	0.564	0.485	0.481	0.510
Maximum	1.00	0.865	0.851	0.852	0.879	0.881
MAE		0.120	0.123	0.121	0.118	0.123
RMSE		0.174	0.175	0.174	0.169	0.172
Log likelihood		144	143.28	149.26	148.11	151.71

**Fig. 1:**
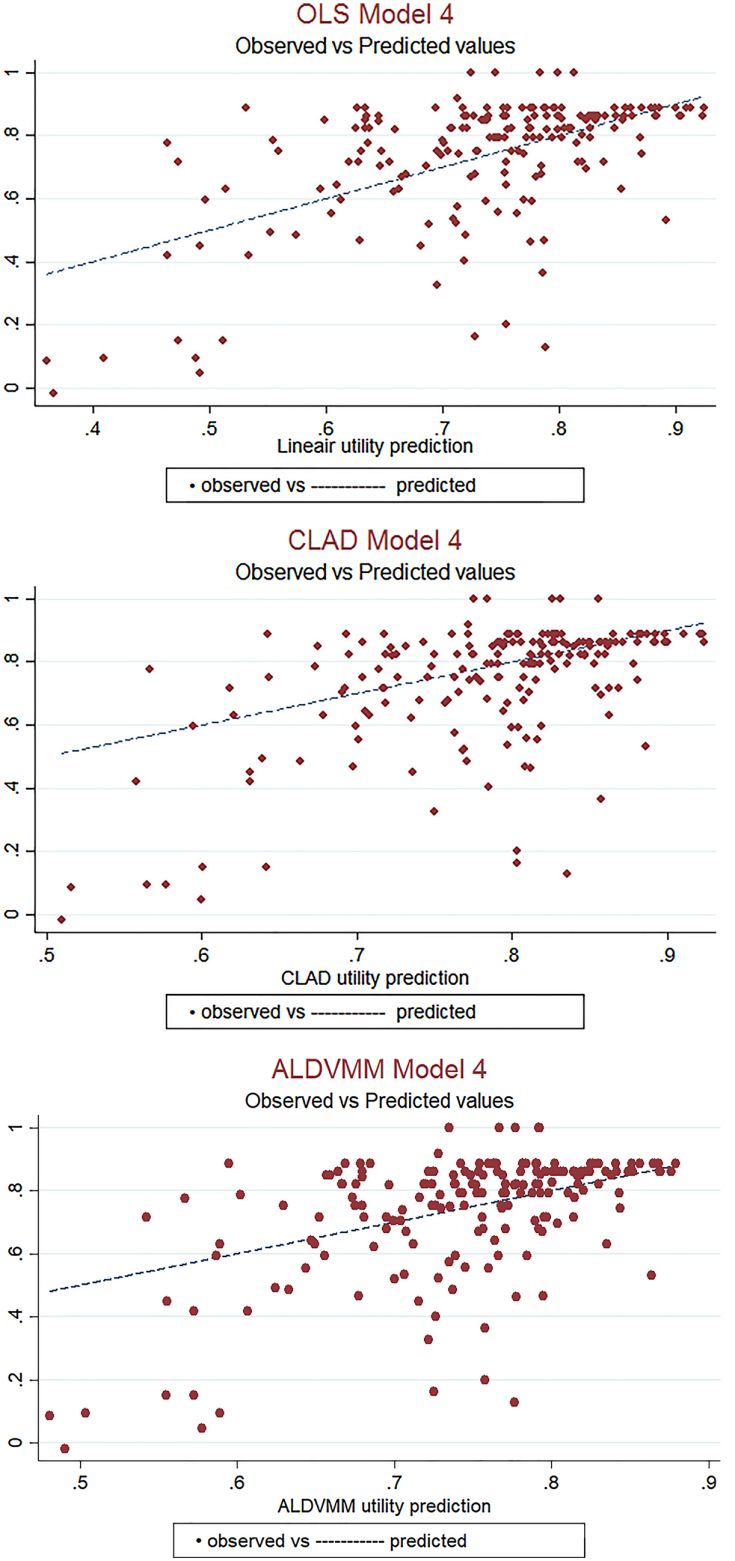
Scatter plots of observed vs predicted EQ-5D-5L utility values for Model 4 (OLS, CLAD, ALDVMM)

The regression coefficients for all models are reported in Supplementary Table 1, 2, and 3. The predicted EQ-5D utilities nearly reached the value of 1 (full health), of which CLAD model 4 was closest to 1 with maximum values of 0.940. Figure 1 shows scatter plots from the observed and predicted utility values for model 4 from all three mapping models. The OLS model shows that the prediction is good at the upper end of the EQ-5D-5L, but worsens when the QoL is at the lower end. The CLAD model shows a good prediction for the higher QoL scores (> 0.7), where the predicted values are equal to the expected values in some cases. However, when the QoL is at the lower end of EQ-5D-5L, the prediction is worse. A large proportion of the observations are present below the truncation point; 0.7 and 0.9. The ALDVMM tends to underestimate good health and overestimate poor health, but observations near the mean are well predicted.

When assessing the goodness of fit results from the validation analysis, by the constructed models from the estimation data set, OLS model 5 showed the lowest MAE of 0.124 and the lowest RMSE of 0.165 of all OLS mapping functions. The CLAD model 4 reported the lowest MAE of 0.124 and RMSE 0.169 of all CLAD mapping functions. The ALDVMM model 4 reported the lowest MAE of 0.128 and RMSE of 0.172 of all mixture functions. These goodness of fit results from the validation analysis are reported in Table 5.

**Table 5:** Summary of observed and predicted values for all models in the external validation dataset (*N* = 84)

	Observed EQ-5D utility	Predicted EQ-5D utilities				
		Total IBS-QoL score	Total IBS-QoL score + IBS-SSS + age	Dysphoria score + Body image score	Total IBS-QoL score + squared IBS-SSS	Dysphoria score + Body image score + squared IBS-SSS + age
		OLS Model 1	OLS Model 2	OLS Model 3	OLS Model 4	OLS Model 5
Mean (SE)	0.72 (0.023)	0.692 (0.111)	0.691 (0.012)	0.699 (0.011)	0.693 (0.014)	0.698 (0.013)
Minimum	− 0.01	0.422	0.410	0.424	0.400	0.412
Maximum	1.00	0.901	0.991	0.884	0.955	0.916
MAE		0.135	0.132	0.125	0.129	0.124
RMSE		0.178	0.173	0.169	0.171	0.165

		CLAD Model 1	CLAD Model 2	CLAD Model 3	CLAD Model 4	CLAD Model 5
Mean (SE)	0.72 (0.023)	0.767 (0.007)	0.742 (0.009)	0.754 (0.009)	0.723 (0.012)	0.776 (0.010)
Minimum	− 0.01	0.611	0.542	0.529	0.449	0.605
Maximum	1.00	0.888	0.939	0.898	0.964	0.889
MAE		0.134	0.128	0.125	0.124	0.132
RMSE		0.189	0.179	0.178	0.169	0.171

		ALDVMM Model 1	ALDVMM Model 2	ALDVMM Model 3	ALDVMM Model 4	ALDVMM Model 5
Mean (SE)	0.72 (0.023)	0.716 (0.008)	0.717 (0.007)	0.721 (0.008)	0.716 (0.010)	0.717 (0.006)
Minimum	− 0.01	0.537	0.580	0.527	0.510	0.536
Maximum	1.00	0.855	0.869	0.852	0.904	0.887
MAE		0.134	0.136	0.131	0.128	0.129
RMSE		0.181	0.182	0.178	0.172	0.173

Given the ease and straightforwardness of the algorithm, the good prediction of the mean and minimum/maximum and a high adjusted/pseudo *R*^2^, model 4 was identified as most appropriate model. Given the lower MAE/RMSE for CLAD model 4 compared to OLS Model 4 and ALDVMM model 4 in the validation sample, the best mapping function would be CLAD model 4, i.e., EQ-5D utility estimate based on total IBS-QoL score + squared IBS-SSS.

## Discussion

This is the first study to present an algorithm to predict utility values in IBS patients from the condition-specific IBS-QoL questionnaire. Results of our mapping approach showed that CLAD model 4 containing the total IBS-QoL score and the squared IBS-SSS score is the most appropriate model to enable prediction of health state utilities. This algorithm was chosen because of its simplicity; the low MAE/RMSE; and the small range to the predicted mean, minimum, and maximum. The mapping from the IBS-QoL to the EQ-5D-5L provides utility scores that can be converted into QALY which is increasingly important in the current health society where economic evaluations are necessary to design reimbursement rules for drugs and medical services.

The reported mean IBS-QoL score in our study from 283 patients was 71.1. These results are similar to those reported in other IBS studies (baseline). In literature, IBS-QoL scores vary between 61.4 and 71.2 [3, 12, 15, 33, 50, 51]. Therefore, our patients’ sample used to derive and validate mapping algorithm covers the most commonly observed IBS-QoL data in clinical practice. Subdomains “Sexual Function” and “Relationships” were least affected in our cohorts in total QoL score. This finding is also in line with earlier studies [33, 51–53]. Patients in our cohorts were most affected by the scores on the subdomain “Food Avoidance” (estimation set 58.5; validation set 47.9). This finding was also confirmed by other studies in IBS patients [33, 51]. In both datasets, the second most affected subdomain was “Health Worry” (estimation set 70.19; validation set 64.19), which reflects the impact of IBS on a psychological level.

The total IBS-QoL score in the validation set was lower than reported in the estimation set (66.88 and 73.02, respectively). This is probably caused by the higher prevalence of moderate depression and mild and moderate anxiety among the IBS patients in the validation set due to offering psychological therapies in this trial. The domains, “dysphoria” and “body image,” of the IBS-QoL, were strongly correlated with the EQ-5D-5L total utility score which highlights the relevance of these domains for IBS patients. Other disease-specific domains such as “food avoidance,” “social reaction,” “sexual,” and “relationships” were less correlated with the total EQ-5D-5L scores and are not represented in the generic questionnaire EQ-5D-5L. Still, these domains are specific and important for the psychological well-being among IBS patients [53]. The overall known-group validity of the IBS-QoL and EQ-5D-5L was quite similar. The IBS-QoL had a greater discriminatory power with regard to age and gender and the severity of symptoms (IBS-SSS). But the EQ-5D-5L had a favorable discriminative power with regard to the presence of anxiety. Both questionnaires showed comparable discriminative power with regard to BMI and the presence of depression. Therefore, our initial hypothesis has to be rejected because the IBS-QoL is not more sensitive to discriminate between disease characteristics compared to the EQ-5D-5L. However, the condition-specific questionnaire IBS-QoL could be more favorable when different aspects of the disease are required to be addressed during a clinical study.

Other condition-specific measures intended for patients who suffer from epilepsy and cancer had a similar sensitivity in comparison to the general EQ-5D [54, 55]. However, in studies involving patients with asthma and urinary incontinence, construct validity of EQ-5D was not as strong as the condition-specific measures [56, 57].

For the final mapping algorithm, we not only included age but also the symptom severity score (IBS-SSS). According to the ISPOR guidelines, including covariates, such as sociodemographic variables and disease characteristics, should be explored to avoid mis-specification of the model [27]. The prediction of the utility values will be more accurate in that way. A recent review of mapping studies showed that age was included in 51% in the algorithm and gender was included in 55% [44]. Clinical measures, such as BMI, were included in the analysis in only 20% of the reports. When performing a mapping study, inclusion of covariates in the algorithm should be explored more extensively in the future to enhance performance.

This is the first study to enable the estimation of utility values from IBS-specific questionnaire scores. A strength of this study includes the applicability to other study IBS populations. The current study population was representable for IBS populations in general because our IBS population have comparable basic patient characteristics (i.e., age, gender) and includes the full range of IBS patient disease severity (range 44–445) [3, 58, 59]. The mean IBS symptom severity score of 278.17 in this study is similar to previous studies (range 259.45–290) [51, 58, 60, 61]. The two data sets used had similar inclusion criteria and the population had similar baseline characteristics, which facilitates the development of a valid mapping approach. Another strength of this study is that a different data set was used for external validation of the models and the model performance was reported by assessing the MAE and the RMSE [42].

A limitation of the present study is that our predicted EQ-5D-5L utilities did not capture the full range of observed EQ-5D-5L utilities. The overprediction of the lowest utilities and the under-prediction of the highest utilities may result in an underestimation of the utility gain. This is a general problem with mapping studies, especially when using linear regression [42, 62]. Therefore, the model fit of both CLAD and ALDVMM outperformed OLS functions. The CLAD Model 4 performed slightly better than ALDVMM Model 4, containing the lowest MAE/RMSE. The big proportion of observations in our dataset was between 0.7 and 0.9. This is below the truncation point, which is an important feature of the ALDVMM, and could be an explanation for the fact that the CLAD model 4 performed better. ALDVMM could be a good option when data are differently distributed than in our dataset.

Furthermore, our algorithm is not directly applicable for usage in trial-based economic evaluations when a comparison with EQ-5D-3L data is requested. However, it is possible to use this data to generate 5L data by conducting a mapping function online [63].

In conclusion, this study investigated a mapping approach where the condition-specific questionnaire IBS-QoL was estimated to EQ-5D-5L utility values. This algorithm is useful for modeling studies in which only the IBS-QoL is included and in trial-based economic evaluations to estimate QALYs. Including a clinical measure in the model, such as the severity score of the disease (IBS-SSS), will improve performance of the algorithm to predict utility values.

## Supplementary Information

Below is the link to the electronic supplementary material.

Supplementary file1 (PDF 144 kb)

Supplementary file2 (PDF 151 kb)

Supplementary file3 (PDF 353 kb)

## Data Availability

All authors confirm that the data supporting the findings of this study are available within the article and its supplementary materials.
