# Supplementary material for: The estimation of a preference-based single index for the IBS-QoL by mapping to the EQ-5D-5L in patients with irritable bowel syndrome
Source: Qual Life Res. 2021 Sep 21;31(4):1209–21. doi: 10.1007/s11136-021-02995-y (PMC8960586; doi:10.1007/s11136-021-02995-y)
Supplement: Supplementary file 2 — Supplementary file2 (PDF 151 kb) [file 11136_2021_2995_MOESM2_ESM.pdf]

Title manuscript: The estimation of a preference-based single index for the IBS-QoL by mapping to the EQ-5D-5L in patients with irritable bowel syndrome.

Journal: Quality of Life Research

Authors: Rosel Sturkenboom MD[1,2], Daniel Keszthelyi MD PhD[1], Lloyd Brandts PhD[2], Zsa Zsa R M Weerts MD[1], Johanna T W Snijkers MD[1], Ad A M Masclee Prof MD PhD[1], Brigitte A B Essers PhD[2]

[1] Division of Gastroenterology-Hepatology, Department of Internal Medicine, NUTRIM School for Nutrition and Translational Research in Metabolism, Maastricht University Medical Center, Maastricht, The Netherlands.

[2] Department of Clinical Epidemiology and Medical Technology Assessment, CAPHRI Care and Public Health Research Institute, Maastricht University Medical Center, Maastricht, The Netherlands.

E-mail address of corresponding author: rosel.sturkenboom@mumc.nl

Supplementary table 2

Mapping equations from IBS-QoL to EQ-5D-5L score using CLAD regression

	Model 1 Total IBS-QoL score		*Model 2 Total IBS-QoL score + IBS-SSS + age*		*Model 3 Two domains IBS-QoL: Dysphoria score + Body image score*		*Model 4 Total IBS-QoL score + Squared IBS-SSS score*		*Model 5 Dysphoria score + Body image score + squared IBS-SSS + age*	
**Type of analysis**	**Clad**		**Clad**		**Clad**		**Clad**		**Clad**	
	Coeff.	SE	Coeff.	SE	Coeff.	SE	Coeff.	SE	Coeff.	SE
Constant	0.408	0.114	0.511	0.114	0.451	0.108	0.471	0.099	0.482	0.098
IBS-QoL total score	0.005	0.001	0.005	0.001			0.005	0.001		
IBS-SSS score			-0.0002	0.0001						
Age			-0.001	0.0009					-0.001	0.0008
IBS-QoL domain Dysphoria score					0.004	0.001			0.004	0.001
IBS-QoL domain Body Image score					0.001	0.001			0.001	0.001
Squared IBS-SSS score							-4.83E-7	3.59e-7	3.75e-07	3.11e-07

Coeff. = Coefficients, determined by CLAD analysis in STATA. SE: Standardized Error. IBS-QoL total score: Score between 0-100. IBS-SSS score: score between 0 and 500 defining the severity of IBS.
