# Supplementary material for: The estimation of a preference-based single index for the IBS-QoL by mapping to the EQ-5D-5L in patients with irritable bowel syndrome
Source: Qual Life Res. 2021 Sep 21;31(4):1209–21. doi: 10.1007/s11136-021-02995-y (PMC8960586; doi:10.1007/s11136-021-02995-y)
Supplement: Supplementary file 3 — Supplementary file3 (PDF 353 kb) [file 11136_2021_2995_MOESM3_ESM.pdf]

Title manuscript: The estimation of a preference-based single index for the IBS-QoL by mapping to the EQ-5D-5L in patients with irritable bowel syndrome.

Journal: Quality of Life Research

Authors: Rosel Sturkenboom MD[1,2], Daniel Keszthelyi MD PhD[1], Lloyd Brandts PhD[2], Zsa Zsa R M Weerts MD[1], Johanna T W Snijkers MD[1], Ad A M Masclee Prof MD PhD[1], Brigitte A B Essers PhD[2]

[1] Division of Gastroenterology-Hepatology, Department of Internal Medicine, NUTRIM School for Nutrition and Translational Research in Metabolism, Maastricht University Medical Center, Maastricht, The Netherlands.

[2] Department of Clinical Epidemiology and Medical Technology Assessment, CAPHRI Care and Public Health Research Institute, Maastricht University Medical Center, Maastricht, The Netherlands.

E-mail address of corresponding author: rosel.sturkenboom@mumc.nl

Supplementary table 3

Mapping equations from IBS-QoL to EQ-5D-5L score using ALDVM models

Model 1

N_NLEQ5D5L_0	Coef.	Std. Err.	z	P>|z|	[95% Conf.	Interval]
Comp_1						
N_QOL_totalscore_0	.0000188	.0003018	0.06	0.950	-.0005728	.0006104
_cons	.8745529	.0223461	39.14	0.000	.8307554	.9183504
Comp_2						
N_QOL_totalscore_0	.0015819	.0001953	8.10	0.000	.0011991	.0019646
_cons	.7303442	.0137961	52.94	0.000	.7033044	.7573841
Comp_3						
N_QOL_totalscore_0	.0084149	.0015697	5.36	0.000	.0053384	.0114914
_cons	-.0001242	.1135621	-0.00	0.999	-.2227019	.2224535
Comp_4						
N_QOL_totalscore_0	.0042495	.0005319	7.99	0.000	.0032071	.0052919
_cons	.470006	.0404947	11.61	0.000	.3906379	.5493742
Prob_C1						
_cons	-.7988692	.375672	-2.13	0.033	-1.535173	-.0625656
Prob_C2						
_cons	-.7903465	.4026044	-1.96	0.050	-1.579437	-.0012563
Prob_C3						
_cons	.073574	.289447	0.25	0.799	-.4937318	.6408798
/lns_1	-4.197175	.1912816	-21.94	0.000	-4.57208	-3.82227
/lns_2	-4.59725	.2146898	-21.41	0.000	-5.018034	-4.176466
/lns_3	-1.550841	.0966175	-16.05	0.000	-1.740208	-1.361474
/lns_4	-3.176991	.1824914	-17.41	0.000	-3.534667	-2.819314
sigma1	.015038	.0028765			.0103364	.0218781
sigma2	.0100795	.002164			.0066175	.0153527
sigma3	.2120695	.0204896			.1754839	.2562827
sigma4	.041711	.0076119			.0291685	.0596468
pi1	.1509586	.0457759			.0811292	.2636463
pi2	.1522506	.0479998			.0797356	.2712739
pi3	.361206	.0521542			.2663636	.4682638
pi4	.3355848	.0578582			.2221847	.4489848

Model 2

N_NLEQ5D5L_0	Coef.	Std. Err.	z	P>|z|	[95% Conf.	Interval]
Comp_1						
N_QOL_totalscore_0	.0006314	.0003847	1.64	0.101	-.0001226	.0013854
N_SSS_Eindscore_0	-.0000559	.000079	-0.71	0.479	-.0002108	.0000989
N_LEEFTIJD_SCREENING	.0003033	.0003325	0.91	0.362	-.0003483	.000955
_cons	.8158511	.0448087	18.21	0.000	.7280278	.9036745
Comp_2						
N_QOL_totalscore_0	.0044806	.0017009	2.63	0.008	.0011469	.0078144
N_SSS_Eindscore_0	-.0008834	.0004141	-2.13	0.033	-.0016951	-.0000717
N_LEEFTIJD_SCREENING	-.0020032	.0029254	-0.68	0.493	-.0077368	.0037303
_cons	.3346758	.1909066	1.75	0.080	-.0394943	.7088459
Comp_3						
N_QOL_totalscore_0	.0037621	.0008812	4.27	0.000	.002035	.0054892
N_SSS_Eindscore_0	-.000677	.0002355	-2.87	0.004	-.0011386	-.0002154
N_LEEFTIJD_SCREENING	.0009462	.0011221	0.84	0.399	-.0012531	.0031455
_cons	.4824217	.0917241	5.26	0.000	.3026457	.6621977
Comp_4						
N_QOL_totalscore_0	.0048154	.0006595	7.30	0.000	.0035229	.006108
N_SSS_Eindscore_0	-.0000119	.0001199	-0.10	0.921	-.0002469	.0002231
N_LEEFTIJD_SCREENING	-.001631	.0007988	-2.04	0.041	-.0031965	-.0000654
_cons	.4859942	.0723942	6.71	0.000	.3441041	.6278843
Prob_C1						
_cons	-.2671206	.3125627	-0.85	0.393	-.8797323	.3454911
Prob_C2						
_cons	-1.500278	.3521834	-4.26	0.000	-2.190544	-.8100108
Prob_C3						
_cons	-1.194805	.3717771	-3.21	0.001	-1.923474	-.466135
/lns_1	-3.542181	.1635771	-21.65	0.000	-3.862786	-3.221576
/lns_2	-2.225104	.2644117	-8.42	0.000	-2.743341	-1.706866
/lns_3	-3.002799	.2428768	-12.36	0.000	-3.478829	-2.526769
/lns_4	-2.908221	.1343496	-21.65	0.000	-3.171541	-2.6449
sigma1	.0289501	.0047356			.0210094	.0398921
sigma2	.1080562	.0285713			.064355	.1814334
sigma3	.0496479	.0120583			.0308435	.0799168
sigma4	.0545727	.0073318			.0419389	.0710124
pi1	.3341087	.0601875			.2279561	.4602263
pi2	.0973497	.0284185			.0541212	.1689392
pi3	.1321295	.0389786			.0725284	.2286346
pi4	.4364121	.0654839			.3080661	.5647582

Model 3

N_NLEQ5D5L_0	Coef.	Std. Err.	z	P>|z|	[95% Conf.	Interval]
Comp_1						
N_QOL_dysphoriascore_0	-.0005819	.000115	-5.06	0.000	-.0008074	-.0003564
N_QOL_bodyimagescore_0	-.0000496	.0001116	-0.44	0.657	-.0002683	.0001692
_cons	.9170211	.0102278	89.66	0.000	.896975	.9370672
Comp_2						
N_QOL_dysphoriascore_0	.0011631	.000322	3.61	0.000	.0005321	.0017941
N_QOL_bodyimagescore_0	.0008845	.0003775	2.34	0.019	.0001447	.0016243
_cons	.6758889	.0239743	28.19	0.000	.6289001	.7228777
Comp_3						
N_QOL_dysphoriascore_0	.0041291	.0032791	1.26	0.208	-.0022978	.010556
N_QOL_bodyimagescore_0	-.0007499	.0037602	-0.20	0.842	-.0081198	.0066199
_cons	.0353644	.1584959	0.22	0.823	-.2752819	.3460106
Comp_4						
N_QOL_dysphoriascore_0	.002492	.0008917	2.79	0.005	.0007443	.0042396
N_QOL_bodyimagescore_0	.0049927	.0010213	4.89	0.000	.0029911	.0069944
_cons	.1497876	.076893	1.95	0.051	-.0009198	.3004951
Prob_C1						
_cons	-1.283383	.3513122	-3.65	0.000	-1.971942	-.5948238
Prob_C2						
_cons	-.1486043	.2744568	-0.54	0.588	-.6865298	.3893212
Prob_C3						
_cons	-1.804801	.5771771	-3.13	0.002	-2.936047	-.6735544
/lns_1	-5.028607	.3262587	-15.41	0.000	-5.668062	-4.389152
/lns_2	-3.368456	.1562448	-21.56	0.000	-3.67469	-3.062222
/lns_3	-2.038223	.4138256	-4.93	0.000	-2.849306	-1.22714
/lns_4	-2.215607	.1348761	-16.43	0.000	-2.479959	-1.951254
sigma1	.0065479	.0021363			.0034546	.0124113
sigma2	.0344428	.0053815			.0253573	.0467836
sigma3	.13026	.0539049			.0578845	.2931298
sigma4	.1090873	.0147133			.0837467	.1420957
pi1	.1202936	.0367355			.0647648	.2126091
pi2	.3741716	.0609985			.2640711	.4990479
pi3	.0714157	.035977			.0258704	.1821514
pi4	.4341191	.0601545			.3162184	.5520197

Model 4

N_NLEQ5D5L_0	Coef.	Std. Err.	z	P>|z|	[95% Conf.	Interval]
Comp_1						
N_QOL_totalscore_0	.0000459	.0002847	0.16	0.872	-.0005122	.000604
Squared_IBSSSS_score	-9.98e-09	7.97e-08	-0.13	0.900	-1.66e-07	1.46e-07
_cons	.8686046	.0245751	35.34	0.000	.8204382	.916771
Comp_2						
N_QOL_totalscore_0	.006668	.0014618	4.56	0.000	.003803	.0095329
Squared_IBSSSS_score	-1.99e-06	5.91e-07	-3.36	0.001	-3.14e-06	-8.28e-07
_cons	.3048719	.1311956	2.32	0.020	.0477333	.5620106
Comp_3						
N_QOL_totalscore_0	.0016212	.0004181	3.88	0.000	.0008018	.0024406
Squared_IBSSSS_score	-3.77e-07	1.74e-07	-2.16	0.031	-7.18e-07	-3.53e-08
_cons	.7440232	.0416033	17.88	0.000	.6624822	.8255643
Comp_4						
N_QOL_totalscore_0	.0042949	.0003908	10.99	0.000	.0035291	.0050608
Squared_IBSSSS_score	-5.37e-07	1.63e-07	-3.28	0.001	-8.57e-07	-2.16e-07
_cons	.499726	.0336493	14.85	0.000	.4337746	.5656775
Prob_C1						
_cons	-.0515761	.3618687	-0.14	0.887	-.7608258	.6576736
Prob_C2						
_cons	.6374391	.3091973	2.06	0.039	.0314235	1.243455
Prob_C3						
_cons	-.1170029	.441314	-0.27	0.791	-.9819625	.7479567
/lns_1	-4.141698	.156953	-26.39	0.000	-4.44932	-3.834076
/lns_2	-1.666758	.0912376	-18.27	0.000	-1.845581	-1.487936
/lns_3	-3.817968	.2821738	-13.53	0.000	-4.371019	-3.264918
/lns_4	-3.741095	.2098454	-17.83	0.000	-4.152385	-3.329806
sigma1	.0158958	.0024949			.0116865	.0216213
sigma2	.1888583	.017231			.1579335	.2258383
sigma3	.0219724	.0062			.0126384	.0382001
sigma4	.0237281	.0049792			.0157269	.0358001
pi1	.2007488	.047818			.1228469	.3105607
pi2	.399842	.0479099			.3105752	.4962963
pi3	.1880349	.0554494			.1020503	.320601
pi4	.2113743	.0499524			.1134693	.3092792

Model 5

N_NLEQ5D5L_0	Coef.	Std. Err.	z	P>|z|	[95% Conf.	Interval]
Comp_1						
N_QOL_dysphoriascore_0	.0008095	.0002873	2.82	0.005	.0002465	.0013726
N_QOL_bodyimagescore_0	.0008299	.0002968	2.80	0.005	.0002481	.0014117
Squared_IBSSSS_score	1.83e-08	1.35e-07	0.14	0.892	-2.46e-07	2.83e-07
N_LEEFTIJD_SCREENING	-.0000816	.0003741	-0.22	0.827	-.0008149	.0006517
_cons	.7203127	.0334533	21.53	0.000	.6547454	.7858799
Comp_2						
N_QOL_dysphoriascore_0	.0016245	.0005256	3.09	0.002	.0005943	.0026546
N_QOL_bodyimagescore_0	.0030349	.0005886	5.16	0.000	.0018812	.0041886
Squared_IBSSSS_score	-1.09e-06	2.28e-07	-4.78	0.000	-1.53e-06	-6.42e-07
N_LEEFTIJD_SCREENING	-.0041577	.0006712	-6.19	0.000	-.0054733	-.0028421
_cons	.6180535	.0582951	10.60	0.000	.5037972	.7323099
Comp_3						
N_QOL_dysphoriascore_0	-.0001995	.0019624	-0.10	0.919	-.0040458	.0036468
N_QOL_bodyimagescore_0	.0029985	.0023042	1.30	0.193	-.0015176	.0075146
Squared_IBSSSS_score	-3.72e-06	9.38e-07	-3.96	0.000	-5.55e-06	-1.88e-06
N_LEEFTIJD_SCREENING	-.0032107	.0023475	-1.37	0.171	-.0078118	.0013904
_cons	.4602395	.1310278	3.51	0.000	.2034297	.7170493
Comp_4						
N_QOL_dysphoriascore_0	.002216	.0009035	2.45	0.014	.0004451	.0039869
N_QOL_bodyimagescore_0	.0003956	.0015278	0.26	0.796	-.0025988	.0033899
Squared_IBSSSS_score	-3.39e-06	5.84e-07	-5.81	0.000	-4.53e-06	-2.25e-06
N_LEEFTIJD_SCREENING	.0035879	.0026005	1.38	0.168	-.0015089	.0086847
_cons	.5415439	.095222	5.69	0.000	.3549122	.7281755
Prob_C1						
_cons	.939732	.2758325	3.41	0.001	.3991103	1.480354
Prob_C2						
_cons	.2276444	.3204753	0.71	0.477	-.4004757	.8557644
Prob_C3						
_cons	-1.469592	.4699436	-3.13	0.002	-2.390664	-.5485192
/lns_1	-3.224936	.0993751	-32.45	0.000	-3.419707	-3.030164
/lns_2	-3.306219	.1707842	-19.36	0.000	-3.64095	-2.971488
/lns_3	-2.794877	.3660135	-7.64	0.000	-3.51225	-2.077504
/lns_4	-2.45808	.2144867	-11.46	0.000	-2.878466	-2.037693
sigma1	.0397583	.003951			.032722	.0483077
sigma2	.0366545	.00626			.0262274	.051227
sigma3	.0611224	.0223716			.0298297	.1252425
sigma4	.0855992	.0183599			.056221	.130329
pi1	.5072981	.053683			.4032931	.6106752
pi2	.2488901	.0483327			.1664119	.3548452
pi3	.045594	.0171066			.0216299	.0935688
pi4	.1982179	.0415872			.1167086	.2797272
